# Study protocol: a randomised controlled trial on the clinical effects of levothyroxine treatment for subclinical hypothyroidism in people aged 80 years and over

**DOI:** 10.1186/s12902-018-0285-8

**Published:** 2018-09-19

**Authors:** R. S. Du Puy, I. Postmus, D. J. Stott, M. R. Blum, R. K. E. Poortvliet, W. P. J. Den Elzen, R. P. Peeters, B. C. van Munster, B. H. R. Wolffenbuttel, R. G. J. Westendorp, P. M. Kearney, I. Ford, S. Kean, C. M. Messow, T. Watt, J. W. Jukema, O. M. Dekkers, J. W. A. Smit, N. Rodondi, J. Gussekloo, S. P. Mooijaart

**Affiliations:** 1 Department of Public Health and Primary Care, Leiden University Medical Center, Leiden, the Netherlands; 2 Department of Gerontology and Geriatrics (C7-Q), Leiden University Medical Center, PO Box 9600, 2300 RC Leiden, The Netherlands; 3 Institute for Evidence-based Medicine in Old age, Leiden, the Netherlands; 4 Geriatric Medicine, Institute of Cardiovascular and Medical Sciences, University of Glasgow, Glasgow, UK; 5 Department of General Internal Medicine, Bern University Hospital, University of Bern, Bern, Switzerland; 6 Department of Clinical Chemistry and Laboratory Medicine, Leiden University Medical Center, Leiden, the Netherlands; 7 Department of Internal Medicine, Erasmus University Medical Centre, Rotterdam, the Netherlands; 8 Department of Internal Medicine, Academic Medical Center, Amsterdam, the Netherlands; 9 Department of Endocrinology, University Medical Center Groningen, University of Groningen, Groningen, the Netherlands; 10 Department of Public Health, University of Copenhagen, Copenhagen, Denmark; 11 Center for Healthy Aging, University of Copenhagen, Copenhagen, Denmark; 12 Department of Epidemiology and Public Health, University College Cork, Cork, Ireland; 13 Robertson Centre for Biostatistics, University of Glasgow, Glasgow, UK; 14 Department of Internal Medicine, Copenhagen University Hospital Herlev, Gentofte, Denmark; 15 Department of Cardiology, Leiden University Medical Center, Leiden, the Netherlands; 16 Department of Endocrinology and metabolic disorders, Leiden University Medical Center, Leiden, the Netherlands; 17 Radboud University Medical Center, Nijmegen, the Netherlands; 18 Institute of Primary Health Care (BIHAM), University of Bern, Bern, Switzerland

**Keywords:** Subclinical hypothyroidism, 80-plus, Randomised controlled trial, Quality of life, Levothyroxine

## Abstract

**Background:**

Subclinical hypothyroidism is common in older people and its contribution to health and disease needs to be elucidated further. Observational and clinical trial data on the clinical effects of subclinical hypothyroidism in persons aged 80 years and over is inconclusive, with some studies suggesting harm and some suggesting benefits, translating into equipoise whether levothyroxine therapy provides clinical benefits. This manuscript describes the study protocol for the Institute for Evidence-Based Medicine in Old Age (IEMO) 80-plus thyroid trial to generate the necessary evidence base.

**Methods:**

The IEMO 80-plus thyroid trial was explicitly designed as an ancillary experiment to the Thyroid hormone Replacement for Untreated older adults with Subclinical hypothyroidism randomised placebo controlled Trial (TRUST) with a near identical protocol and shared research infrastructure. Outcomes will be presented separately for the IEMO and TRUST 80-plus groups, as well as a pre-planned combined analysis of the 145 participants included in the IEMO trial and the 146 participants from the TRUST thyroid trial aged 80 years and over.

The IEMO 80-plus thyroid trial is a multi-centre randomised double-blind placebo-controlled parallel group trial of levothyroxine treatment in community-dwelling participants aged 80 years and over with persistent subclinical hypothyroidism (TSH ≥4.6 and ≤ 19.9 mU/L and fT4 within laboratory reference ranges). Participants are randomised to levothyroxine 25 or 50 micrograms daily or matching placebo with dose titrations according to TSH levels, for a minimum follow-up of one and a maximum of three years.

Primary study endpoints: hypothyroid physical symptoms and tiredness on the thyroid-related quality of life patient-reported outcome (ThyPRO) at one year. Secondary endpoints: generic quality of life, executive cognitive function, handgrip strength, functional ability, blood pressure, weight, body mass index, and mortality. Adverse events will be recorded with specific interest on cardiovascular endpoints such as atrial fibrillation and heart failure.

**Discussion:**

The combined analysis of participants in the IEMO 80-plus thyroid trial with the participants aged over 80 in the TRUST trial will provide the largest experimental evidence base on multimodal effects of levothyroxine treatment in 80-plus persons to date.

**Trial registration:**

Nederlands (Dutch) Trial Register: NTR3851 (12–02-2013), EudraCT: 2012–004160-22 (17–02-2013), ABR-41259.058.13 (12–02-2013).

**Electronic supplementary material:**

The online version of this article (10.1186/s12902-018-0285-8) contains supplementary material, which is available to authorized users.

## Background

Subclinical hypothyroidism (SCH) is a common aberrant biochemical finding defined as an elevated serum thyroid-stimulating hormone (TSH) and normal circulating thyroid hormone level [1]. SCH is associated with multiple health problems in old age ranging from mild non-specific symptoms such as tiredness and emotional susceptibility to coronary heart disease and decreased physical and cognitive functioning [2].

As 8–18% of those over 65 years are affected and inference from both observational and experimental studies maintain the clinical equipoise whether the merits of levothyroxine treatment outweigh the risks [3], the Thyroid hormone Replacement for Untreated older adults with Subclinical hypothyroidism randomised placebo controlled Trial (TRUST) [4] was designed to resolve this clinical uncertainty. The outcomes of the TRUST trial provided robust information that for community-dwelling persons of 65 years of age and older with SCH, levothyroxine treatment provides no apparent benefits [4].

There are ample data to suggest that thyroid function is mediated by age and that the effects of SCH may be profoundly different in octogenarians and older [3]. Older persons generally require different dosages of levothyroxine to achieve euthyroidism than younger counterparts possibly due to changes in body weight, composition or hormonal status [5] and are at higher risk of adverse effects of overtreatment including cardiovascular events, arrhythmias and fractures [6]. In a large-scale, observational follow-up study among 599 community-dwelling participants aged 85 years and over, increasing levels of TSH were associated with prolonged life span [7]. This association, however, could not be confirmed in a later Individual Patient Data meta-analysis investigating mortality information in 4344 participants with SCH aged 80 years and over [8]. In addition, members of families with exceptional longevity are characterized by slightly higher TSH and slightly lower circulating thyroid hormone levels when compared with the general population [9].

To help resolve this clinical uncertainty of levothyroxine replacement treatment for SCH in older persons, we have performed an randomized controlled trial including participants over 80 years old in the presence of comorbid conditions; the Institute for Evidence-Based Medicine in Old Age (IEMO)80-plus thyroid trial. The TRUST trial was not designed specifically to investigate the effects in 80-plus participants and was consequently inadequately powered for a subgroup analysis in participants aged 80 and over. The IEMO 80-plus thyroid trial was designed jointly with the TRUST trial as an ancillary trial using the same trial infrastructure and protocol to allow a pre-planned, joint analysis of all participants aged 80 and over. This combined endeavour will provide experimental evidence on potential multimodal effects of levothyroxine treatment from the largest sample of 80-plus persons with SCH to date.

Among the specific study objectives are:

1. Does levothyroxine treatment for SCH provide benefits for 80-plus persons with SCH?
2. Are benefits seen across a wide range of outcomes, including health-related quality of life, muscle function, cognition and prevention of cardiovascular disease?
3. Are benefits seen in specific subgroups of people with SCH, including women, and those with mild degrees of SCH (TSH 4.6–10 mU/L)?
4. Are any benefits offset by adverse effects, such as atrial fibrillation or heart failure?

## Methods and design

The IEMO 80-plus thyroid trial was designed as an ancillary randomised double-blind placebo-controlled parallel group trial of levothyroxine for persons over 80 years with subclinical hypothyroidism. From the outset the study was designed jointly and in parallel with the TRUST trial (details provided elsewhere [10]) and both trials share a near identical design and infrastructure including study protocols, standard operating procedures, independent data monitoring and endpoint committees, databases, statisticians and study nurses.

Initially, the IEMO 80-plus thyroid trial aimed to include 450 participants. Additionally, a pre-planned combined analysis with the data from all 80-plus participants from the TRUST trial, resulting in a total of 900 participants in the final pooled analyses, was conceived to maximise statistical power. During the inclusion phase, it became apparent that the proposed target of 450 80-plus participants was unfeasible within the allotted study period (mirroring the experiences of the TRUST trial [10]) and revised power calculations were proposed with the new projected target of 145 IEMO 80-plus trial participants (see Sample size calculation).

Originally the trial was executed in 4 regions of the Netherlands (Leiden University Medical Center, Erasmus University Medical Center, University Medical Center Groningen and the University of Amsterdam). During the inclusion period, in an attempt to maximise the inclusion rate, organisational changes were accepted allowing for inclusion of participants from all locations within the Netherlands, coordinated by the Leiden University Medical Center. Additionally, because the trial infrastructure was already in place for the TRUST trial, additional participants were recruited from the University Hospital Bern in Switzerland.

### Study population

One hundred forty five community-dwelling participants ≥80 years with SCH are recruited. Similar to TRUST, participants are identified from clinical and primary care laboratory databases from all patients having biochemical features consistent with SCH. SCH is defined as persistently elevated TSH levels (≥ 4.6 and ≤ 19.9 mU/L), measured on a minimum of two occasions at least 3 months and no more than 3 years apart prior to enrolment and free thyroxine (fT4) within the laboratory reference range. All participants gave written individual informed consent to participate.

Exclusion criteria

- Participants currently on levothyroxine, antithyroid medication (including carbimazole, methimazole, propylthiouracil and potassium perchlorate), amiodarone or lithium.
- Recent thyroid surgery or radio-iodine therapy (within 12 months).
- Grade IV NYHA heart failure.
- Prior clinical diagnosis of dementia.
- Recent hospitalisation for major illness (within 4 weeks).
- Recent acute coronary syndrome, including myocardial infarction or unstable angina (within 4 weeks).
- Acute myocarditis or acute pancarditis
- Untreated adrenal insufficiency or adrenal disorder
- Terminal illness.
- Patients known to have rare hereditary problem of galactose intolerance.
- Participants who are participating in ongoing RCTs of therapeutic interventions (including clinical trials of investigational medicinal products [CTIMPs])
- Plan to move out of the region in which the trial is being conducted within the next 2 years.

### Intervention

The investigational medicinal products are levothyroxine sodium (T4) as 25 or 50 microgram tablets for oral administration and a matching placebo. All tablets are white and round in shape with the strength imprinted, identically packaged in blisters and packed in plain cardboard cartons to maintain study blinding. Participants are advised to take the suggested dose of study medication once daily half an hour before breakfast.

The intervention group will start with levothyroxine 50 micrograms daily (25 micrograms in participants with < 50 kg body weight or with a history of coronary heart disease) and the control group with matching placebo for six to 8 weeks.

After 6–8 weeks a venous blood sample is taken for TSH assessment. Based on the TSH results, the data centre advises the new dose of study medication or placebo to the clinical investigators.

- If TSH < 0.4 mU/L: the treatment dose is reduced to 25 micrograms levothyroxine in those starting on 50 micrograms; reduced to 0 in those starting on 25 micrograms – effected by giving placebo matching the 25 micrograms dose. These participants will have a further TSH check after 6–8 weeks. If TSH remains < 0.4 mU/L participant will be withdrawn from randomised treatment and referred to usual care.
- If TSH ≥ 0.4 and < 4.6 mU/L: no change to the treatment dose.
- If TSH remains elevated (≥ 4.6 mU/L): 25 micrograms of levothyroxine will be added. Giving a total daily dose of 75 micrograms levothyroxine for those starting on 50 micrograms, or a total daily dose of 50 micrograms levothyroxine for those starting on 25 micrograms.

A maximum of two levothyroxine up-titrations at the start of the trial and one up-titration at 12 and 24 month (± 1 month) intervals with repeated TSH measurements after 6–8 weeks ensure adequate levothyroxine treatment while avoiding potential over-replacement. The maximum possible dose of levothyroxine is 150 micrograms.

A mock titration adopting an adaptive schedule is performed in the placebo group by the data centre. A similar proportion of placebo patients will have up and down titrations of study medication as the intervention group to ensure the number of tablets and assessments is similar in both groups.

Because all thyroid function measurements are available only to the data centre, the clinical investigators remain fully blinded to the treatment allocation process during the trial.

Accountability logs recording the quantities of study medication dispensed to and returned from study participants, batch numbers and expiry dates are available for all study drug movements.

Criteria for discontinuing or modifying allocated study medication:

- If overt biochemical hypothyroidism is identified (TSH > 20 mU/L and/or fT4 below the reference range) a second TSH with fT4 within 2 weeks is requested. Upon confirmation of biochemical hypothyroidism the participant will be withdrawn from the study treatment and referred to the General Practitioner (GP) for usual care.
- If overt biochemical hyperthyroidism is identified (TSH < 0.4 mU/L) in the placebo group, or consecutively in the treatment group despite downtitrations, the participant will be withdrawn from the study treatment and referred to the GP for usual care.
- If for clinical reasons (e.g. major illness) a proposed change in study medication or placebo is deemed inappropriate the algorithm is overridden by the local principal coordinator and no change in study medication takes place.

### Randomisation

Participants are randomised to either the levothyroxine or placebo treatment arm (ratio 1:1) using the randomly permuted block method, stratified by site, sex and starting dose. The data centre (Robertson Centre for Biostatistics, University of Glasgow, Scotland) independently provides the randomisation schedule. Mawdsley Brooks & Co. implements the schedule through identical packaging of levothyroxine and matching placebo tablets.

Patient allocation is conducted via the dedicated trial web portal by the study nurses. When a participant is eligible based on entering the eligibility criteria in an electronic case report form (eCRF) supervised by a medically certified Principle Investigator, a central computer will trigger the decision.

### Blinding

Participants are blinded to treatment allocation by using matching tablets and packaging for levothyroxine and placebo. All study personnel remain blinded for the duration of the trial through remote analysis of laboratory results of TSH in the data centre, ensuring the trial stays double blinded. GPs will remain blinded to treatment allocation and TSH tests unless otherwise required in the event of an emergency medical situation. An Interactive Voice Response System at the data centre allows for individual emergency allocation information to be released to an unblinded study physician through 24-h telephone access. All participants will learn the treatment allocation within 15 working days of receiving the final visit and completing all the data to aid in any further treatment decisions with the GP.

All laboratory tests for TSH and fT4 are performed at the local GP and clinical laboratories. The results in the treatment phase are uploaded to the independent data centre which in turn advises the study site on dose titration through the dedicated trial web portal. The study team remains unaware of the results of the thyroid function testing. Additionally, all cooperating GPs were asked to refrain from additional thyroid function measurements to ensure adequate blinding.

### Data collection

Data collection will be performed by study research nurses at baseline and predetermined follow-up visits at the participant’s home or place of residence. All participants are followed up for a minimum of 1 year with a likely average of 2 years. Participants are reviewed face-to-face by the study nurses at recruitment, study baseline, 6–8 weeks, 12 months, 24 months, 36 months and at the final visit. This personal approach ensures data quality and promotes participant retention and complete follow-up. In addition, interim telephone contact or visits (depending on the desire of the patient) are made by study nurses at 6, 18 and 30 months (depending on total duration of follow-up), including recording of possible cardiovascular and serious adverse events (SAEs). For a timeline of assessments and visits see Table 1.

**Table 1:** Detailed schedule of assessments

Months of follow up	0 visit	6-8 wks	6m	12m	18m	24m	30m	36m	Final^a^ visit
		visit	call/visit	visit	call/visit	visit	call/visit	visit	
Participant characteristics & medical history	x								
Weight, height, waist circumference and BMI	x			x					x
Concomitant medication	x	x		x		x		x	x
Home support	x								x
Safety and monitoring									
Morbidity, mortality, hospitalisation and GP contacts		x	x	x	x	x	x	x	x
Serious Adverse Events		x	x	x	x	x	x	x	x
Single-lead ECG (for AF)	x			x		x		x	x
Drug adherence		x	x	x	x	x	x	x	x
Outcomes									
Thyroid related quality of life (ThyPRO)	x	x		x					x
Generic quality of life (EQ-5D-3L)	x	x		x					x
Cognitive function									
MMSE	x								
Letter Digit Coding Test	x								x
Functional ability									
ADL (BI), IADL (OARS), falls questionnaire	x								x
Handgrip strength & 6-meter gait speed	x			x					x
Blood pressure	x			x					x
Fatal and non-fatal cardiovascular events		x	x	x	x	x	x	x	x
Arthritis complaints									x
Treatment Satisfaction (TSQM vII)									x
Laboratory analysis									
Thyroid function	x	x		x		x		x	x
Haemoglobin	x			x					
Blood samples for biobank	x			x					

^a^the final visit assessments may substitute for any assessment time between 12 and a maximum of 42 months

All study nurses are trained simultaneously on the data to be assessed. All measuring equipment is calibrated before the start and annually thereafter to safeguard reliability and validity. The Data centre will develop and manage a dedicated, anonymised trial web portal, including the electronic case report forms in Dutch and Swiss Standard German. This portal is based on the dedicated trial web portal from the TRUST trial to maximize the homogeneity of data and to allow for pre-planned pooled analysis of the results. Personal information used for trial logistics is collected and stored in a separate electronic study database in accordance with legal and ethical requirements.

Data validation checks give study personnel immediate feedback on missing or out of range values. Logic checks reduce the possibilities of entering invalid data. Database validation checks are run routinely and are tracked and escalated as appropriate. Data will be locked at the end of the study according to preregistered lockdown procedures by the data centre. The data centre will provide the independent data monitoring committee (IDMC) and the authorities with (annual) safety reports on the Data.

### Outcomes

At 6–8 weeks we expect most patients allocated to levothyroxine to be biochemically euthyroid, and at this time point short-term improvements (such as in thyroid-related quality of life) will be assessed. By 1 year the medium-term effects of levothyroxine treatment should emerge (such as muscle function). The long term effects of treatment of SCH will be determined by assessment over the full course of the study, with a mean of 2 years treatment duration.

In the screening phase, results from TSH and fT4 tests, exclusion criteria, informed consent for the screening phase of the study, informed consent for the trial phase of the study are obtained by the study nurses.

During the baseline phase of the study the following data are recorded:

- Participant characteristics: age, sex, ethnicity, information on alcohol and tobacco use, height.
- Any clinical changes that would violate the inclusion or exclusion criteria
- Concomitant drugs used: prescribed medication, over-the-counter non-steroidal anti-inflammatory drugs and aspirin
- History of disease: Cardiovascular disease including history of ischaemic heart disease (angina pectoris or previous myocardial infarction), cerebrovascular disease (ischaemic stroke, transient ischaemic attack) or peripheral vascular disease (intermittent claudication), or any revascularisation procedure for ischaemic vascular disease. History of atrial fibrillation, epilepsy, hypertension, diabetes mellitus or osteoporosis.
- Single lead ECG: to check for atrial fibrillation
- Cognitive function: Mini-mental state examination (MMSE [11]) score as an indicator of general cognitive function. This will not be used as an outcome measure due to insensitivity to change during the trial.
- Home support services: (e.g. home help, meals-on-wheels, district nursing) and home circumstances (e.g. living alone, co-habiting, standard or sheltered housing, or entry to care home)

#### Primary study endpoints

The main study primary endpoints are mean change from baseline scores in thyroid-related quality of life and symptom burden assessed using the hypothyroid symptoms scale score and tiredness symptoms scale score on the thyroid-related quality of life patient-reported outcome (ThyPRO) [12] at 12 months after recruitment. The primary analyses will be done in the 80 years and over group (IEMO and TRUST participants). The results will be compared through subgroup analysis with those in the 79 years and under group (TRUST participants) as a secondary analysis. The ThyPRO is an 85-item patient-reported outcome measure, evaluating symptoms, well-being and function on 85 items summarised in 14 scales, ranging 0–100, with higher scores representing more symptoms or impact of disease. For this study three scales with 19 items are evaluated: Tiredness, Hypothyroid physical symptoms and Hyperthyroid physical symptoms.

#### Secondary study endpoints

- Generic quality of life: EuroQOL EQ-5D-3 L [13] at baseline, 6–8 weeks, 12 months and final follow up.
- Thyroid-related quality of life ThyPRO [12] at baseline, 6–8 weeks and at final follow-up.
- Thyroid-related quality of life: ThyPRO-39 [14] recorded at final follow-up (additional 28 questions).
- Executive cognitive function: Letter Digit Coding Test [LDCT] [15] at baseline and final follow-up.
- Handgrip strength: Jamar hand dynamometer (best of 3 measures in dominant hand) at baseline, 12 months and final follow up.
- Functional ability: Activities of Daily Living (Barthel Index [BI] [16, 17]), Instrumental Activities of Daily Living (Older Americans Resources and Services [OARS] [18]), 6- m gait speed [19], independent living status and falls questionnaire at baseline and final follow up.
- Blood pressure: systolic and diastolic measured at baseline, 12 months and final follow up
- Height, weight, waist circumference and body mass index: recorded at baseline, 12 months and final follow up
- Mortality: all-cause and cardiovascular are requested through national mortality registries
- Fatal and non-fatal cardiovascular events: including acute myocardial infarction, stroke, amputations for peripheral vascular disease and revascularisations for atherosclerotic vascular disease (including for acute coronary syndrome and heart failure hospitalisations).

#### Additional measurements

- Treatment satisfaction with trial medication: Treatment Satisfaction Questionnaire for Medication vII (TSQM [20]) and desire of post-trial medication continuation recorded at final follow up.
- Arthritis: data regarding joints, skeletal functioning and arthritis are recorded through an arthritis questionnaire at final follow up.
- Haemoglobin: measured on a full blood count at baseline and 12 months.

See Table 1 for detailed schedule of assessments.

### Safety

Full details of all Serious Adverse Events (SAEs), Adverse Events (AEs) of special interest (atrial fibrillation, heart failure, fractures, new diagnosis of osteoporosis), study treatment withdrawals and ThyPRO hyperthyroid symptoms are recorded at all visits and telephone contacts. Participants and GPs have 24-h access to an emergency trial phone number operated by a certified physician for the reporting of SAEs.

### Biobank

Blood samples for the IEMO biobank are collected at baseline (40 ml venous blood) and at 12 months (10 ml venous blood). The following 19 aliquots (0.75 ml each) are stored per participant at baseline: 3 EDTA plasma, 1 whole blood, 2 citrated plasma, 1 NaF plasma, 1 buffy coat, 3 heparin plasma, and 8 serum aliquots. The 12 months bloods are stored in four serum 0.75 ml aliquots per participant.

Analyses in the IEMO biobank will be performed in combination with the TRUST biobank. Both biobanks are organised by the same biobank committee. The IEMO biobank will be stored at the Department of Clinical Chemistry of Leiden University Medical Center (LUMC), the Netherlands. The biobank consists of all plasma, serum, and DNA material of all randomised IEMO participants that provide consent for storing biobank material. The Department of Clinical Chemistry of the LUMC is fully accredited (EN ISO 15189:2012) by the Dutch Accreditation Council. The Biobank adheres to all necessary quality assurance standards and legal guidelines.

### Sample size calculation

The total number of participants in all published trials on SCH before 2017 is 450 across 12 studies, including only a small numbers of older people and very heterogeneous endpoints across studies.

We aim to study endpoints that are of particular relevance for the oldest old, including endpoints in those with considerable comorbidity.

Originally, the IEMO 80-plus thyroid trial had set out to analyse 450 participants with SCH aged 80 years and over. Additionally, a pre-planned pooled analysis of 900 participants was agreed upon, of which 450 were recruited directly through this study and a subset over the age of 80 years from the TRUST trial would add another 450 participants, to further increase the statistical power to detect significant changes in this subgroup. The power calculations were based on two main study endpoints:

1. Fatal and non-fatal cardiovascular events.
2. Change in thyroid-related quality of life (ThyPRO Tiredness and Hypothyroid physical symptoms).

Due to several limiting factors including delays in starting the studies, caused by difficulties procuring study medication and matching placebos, it proved impossible to reach this number, similar to the experiences in the TRUST trial [10]. Therefore, in 2015, revised power calculations were proposed (study protocol amendment 8, 04/06/2015) and accepted by the funding agent, sponsor, medical ethical committee (15/07/15) and competent authority (03/07/2015). These revisions detailed the change of primary study endpoint cardiovascular events into a secondary study outcome, accepting the possibility of being underpowered to answer this secondary endpoint. This allowed the power calculations to be revised according to the remaining primary outcome thyroid-related quality of life.

The resulting revised sample-size calculation is based on the pre-planned pooled analysis of one of the co-primary endpoints of thyroid-related quality of life (ThyPRO Hypothyroid physical symptoms and Tiredness scale score). According to previous studies applying the ThyPRO, a study should be adequately powered for at least a difference of 9 points to be clinically meaningful. Using an expected standard deviation of the difference of 26 [21] and a power of 80%, 132 participants are required per trial group adding to a total of 264 participants to be included in the combined 80-plus analyses. For all secondary continuous endpoints this sample size is deemed large enough to provide statistically robust results. For the secondary endpoints on cardiovascular events and mortality the possibility of being underpowered is accepted.

Over a recruitment period of almost 3 years the TRUST trial recruited 737 participants to the trial of which 146 participants were aged 80 and over. Assuming 10% loss to follow-up in both trials a projected 145 additional participants will be recruited in the IEMO trial. The follow up phase of the trial is expected to be complete in May 2018 with one additional month of SAE recording.

### Data analysis

The data centre (Robertson Centre for Biostatistics, Glasgow, ISO 9001/2008 certified) is responsible for writing, implementing and revising a statistical analysis plan that is agreed upon before locking the study database and will have full access to the final study database for the planned analyses. A copy of the statistical analysis plan is appended to this manuscript as Additional file 1. All analyses are based on a modified intention-to-treat principle and the primary time-point for analysis is after 12 months of treatment. The main analyses will be based on the combined IEMO and TRUST 80-plus participants (*n* = 291).

Analyses will be presented separately for:

- the IEMO 80-plus participant cohort (*n* = 145)
- the TRUST 80-plus participant subset (*n* = 146)
- the combined IEMO and TRUST 80-plus participants compared with the TRUST 80-minus participants (*n* = 291 vs *n* = 591)

Summary information for all participants and between the treatment groups will be made available. Similar to the TRUST trial [10], continuous variables measured at baseline and follow-up will be analysed at each time point comparing treatment groups adjusting for stratification variables and baseline levels of the same variable using analysis of covariance. Additionally, repeated measures regression analysis will be performed with regards to the primary time-point and final assessment for each participant. For calculating ThyPRO scores, raw total scores containing valid missing items will be scaled to maintain the maximum possible score. Clinical outcome data will be analysed using time-to-first-event Cox proportional hazards regression analysis in models that contain the randomised treatment allocation and stratification variables as covariates. Treatment effect will be analysed using the Wald-test and corresponding point estimates and 95% confidence intervals for the hazard ratio for treatment will be estimated. The assumption of proportionality of hazards will be tested.

Analysis of the primary outcomes will be performed in the modified intention to treat (ITT) population, based on participants with data available on the outcome of interest. The ITT population will be used for analyses on efficacy and safety. In addition, analyses using mixed effects models and multiple imputations will be used for sensitivity analysis. The per protocol population will also be used for all primary and secondary outcomes as exploratory analyses.

Owing to the intended similarities in study design between the IEMO 80-plus thyroid trial and the TRUST trial, the data allow for a pooled subgroup analysis of the TRUST and IEMO 80-plus participants compared with the TRUST 80-minus participants. Outcome differences between these groups will highlight the additional clinical merits or adverse effects of levothyroxine replacement therapy for older participants aged 80 and over.

Other pre-planned subgroup analyses include: baseline TSH in two groups (< 10/≥10 mIU/L) or in three groups (< 7/7–9.99/≥10 mIU/L), sex (male/female). However, we accept that our study will be underpowered for some of the smaller subgroups, such as male participants, TSH above 10.0 and below 19.9 mIU/L. We should however have sufficient statistical power in the combined analysis to detect beneficial effects in the larger or dominant subgroups, such as female and TSH in the range above 4.6 and below 10.0 mIU/L.

### Monitoring and committees

To secure the highest quality of participant care and safety, the careful titration algorithm avoids the possibility of prolonged periods of levothyroxine over-replacement. Similarly, the system guards against participants developing overt hypothyroidism that might require open-label levothyroxine use.

All SAEs, AEs and AEs of special interest are recorded, notified, assessed, reported, analysed and managed in accordance with the Medicines for Human Use (Clinical Trials) Regulations 2004 and the study protocol. All events are followed up until resolution or stabilization occurs, and are assessed for seriousness, expectedness and causality by the chief investigator. Serious adverse events are reported to the sponsor by thorough recording in the eCRF and to the local accredited Medical Ethics Committee and competent authority. Annually and at the end of the trial 100% study monitoring visits are conducted by independent clinical research associates, in accordance with the Netherlands Federation of University Medical Centres’ report ‘Kwaliteitsborging van Mensgebonden onderzoek’. All important decisions made leading to protocol modifications are communicated to all relevant parties, including the trial registry, ethical committees and competent authorities.

All main decisions for the study were made by the steering group. Its members are: Dr. Simon P. Mooijaart, Dr. Jacobijn Gussekloo, Dr. Olaf M. Dekkers, Dr. Jan Smit, Dr. J.Wouter Jukema, Dr. Anton. J.M. de Craen (Leiden, the Netherlands, Deceased).

Each national site was supervised by a local organising committee and Principle Investigator. For the Netherlands the organising committee was: Dr. Simon P. Mooijaart (PI), Dr. Rosalinde K.E. Poortvliet, Dr. Iris Postmus, Robert S. Du Puy, MSc, Professor Robin. P. Peeters, Professor Bruce. H.R. Wolffenbuttel and Dr. Barbara. C. van Munster. For Switzerland the organising committee was: Professor N. Rodondi (PI) and Dr. Manuel Blum,

An Independent Data Monitoring Committee (IDMC) assesses safety data in order to protect the ethical and safety interests of the participants recruited into the study, while safeguarding, as far as possible, the scientific validity of the study. The IDMC reviews annual safety and efficacy data and may request additional data if considered necessary. The IDMC meets at least once a year and is composed of medical experts and a biostatistician without any involvement in the study as investigators or as study participant care physicians. The committee is empowered to make a recommendation on early stopping when there is overwhelming evidence of benefit for the primary outcome or when it considers there is adequate evidence of harm. The IDMC members are: Professor Gary Ford (Chair; Chief Executive Officer of the Oxford Academic Health Science Network, Oxford), Professor Thompson G Robinson (University Hospitals of Leicester NHS Trust, Department of Cardiovascular Sciences, Leicester Royal Infirmary, Leicester), Professor Colin Dayan (Institute of Molecular and Experimental Medicine, Cardiff University School of Medicine, Heath Park, Cardiff), Professor Kathleen Bennett (Department of Pharmacology and Therapeutics, Trinity Centre for Health Sciences, St James’s Hospital, Dublin).

A study Endpoint Committee, blinded to treatment allocation, provides independent and unbiased review of clinical endpoint events which occur during the study, ensures unified and unambiguous events evaluation practices across the study and compensates for regional diversity in medical practice at the site of endpoint evaluation and classification. All causes of death, stroke, myocardial infarction and heart failure hospitalisations are potential endpoints to be reviewed on the data supplied through the eCRF and if necessary acquired source documentation. The Endpoint Committee members are: Professor Peter Langhorne (Chair; Professor of Stroke Care, Institute of Cardiovascular and Medical Sciences, University of Glasgow), Professor J Wouter Jukema (Vice-chair; Professor of Cardiology, Leiden University Medical Center, The Netherlands), Dr. Tin Hai Collet (Department of Ambulatory Care and Community Medicine, University of Lausanne, Switzerland), Professor Olaf M Dekkers Leiden University Medical Center, The Netherlands) and Dr. Anne Marie O’Flynn (Department of Epidemiology and Public Health, UCC, Ireland).

A TRUST/IEMO Biobank committee supervises the storage and analysis of the biobank samples. Members are: Professor Patricia M. Kearney, Dr. H. Anette van Dorland (Bern, Switzerland), Dr. Wendy P.J. den Elzen.

Each national study site is supervised by a local sponsor, responsible for the oversight of the clinical trial and supplying proper insurance to cover any liabilities during and after the trial arising from trial conduct and participation. The sponsors is not involved in the preparation, or approval of any scientific outputs.

### Dissemination

This study is well suited to promote effective dissemination of the results and implications. Arrangements regarding sharing of data and joint publication are laid down in a Memorandum of Understanding between the TRUST trial and IEMO 80-plus thyroid trial project group. Due to its role as a knowledge centre with an education and research program in the field of ageing, vitality and geriatric medicine, the Leiden Academy on Vitality and Ageing is well placed to play a coordinating role in the dissemination activities. Schildklier Organisatie Nederland, the patient advocacy group, will closely collaborate with the study team to help align the study outputs with the patients and public need.

The Institute for Evidence-based Medicine in Old Age (the Netherlands) is ideally placed to ensure that the results of the study are considered by relevant professionals, and will be included in the leading clinical guidelines. In cooperation with the Cochrane collaboration the results of the trial will be offered for the update of the Cochrane systematic review of treatment of subclinical hypothyroidism, allowing for independent scientific interpretation, placing results in context and maximising understanding of the implication of the trial.

To comply with the general social responsibility associated with clinical research, the trial results will be proactively disseminated to the general public and key public health stakeholders through established media networks.

## Discussion

In the latest Cochrane review of levothyroxine replacement therapy for SCH (12 studies, only 491 participants in total) most studies excluded those who suffered from multimorbidity, none of the studies reported on oldest old separately and two trials excluded those over the age of 80 years [22]. Robust evidence for the potential clinical merits or adverse effects in 80-plus persons with SCH is greatly needed to help guide clinical practice.

The IEMO 80-plus thyroid trial is a representative randomised controlled trial on levothyroxine treatment for SCH, with representative 80-plus persons and a wide range of characteristics and morbidities, studying end-points that are relevant to the older population and clinical practice. The combined analysis of participants in the IEMO 80-plus thyroid trial with those aged over 80 who participated in the TRUST trial will provide the largest experimental evidence base on the multimodal effects of levothyroxine treatment in 80-plus persons to date. Trial results are expected to be publicly disseminated in the fall of 2018.

### Additional files

Additional file 1: Statistical analysis plan. (PDF 114 kb)

Additional file 2: Participant consent form for screening. (PDF 68 kb)

Additional file 3: Participant consent form randomisation. (PDF 83 kb)

## Appendix 1: Patient Information leaflet for screening

**Thyroid hormone replacement for older persons with mild thyroid dysfunction.**

*In the past 3 years blood was drawn at your general practice office or in the hospital. The results showed that you may be eligible for participating in the IEMO 80+ Thyroid Trial. For this reason your general practitioner or hospital specialist sends you this letter and the information leaflet regarding this research study.*

**1. What is the purpose of the research study?**

Thyroid hormone has many important functions in the human body, for example supporting the correct function of the muscles, circulation, the brain and metabolism. When a shortage of thyroid hormone is present, bodily functions may not work optimally.

The results of one of your blood tests from the past suggest that you may have mild thyroid dysfunction (subclinical hypothyroidism). This is when thyroid hormone levels are within the normal laboratory limits, but signs are present that the body is urging the thyroid gland to work harder. This is usually a chance finding. This particular blood test result is common in older persons: of all persons aged 65 years and over 1 in 6 may have subclinical hypothyroidism. It is currently unknown whether it is beneficial to treat subclinical hypothyroidism with artificial thyroid hormone. The IEMO 80+ Thyroid Trial was set out to investigate this problem. The purpose of the IEMO 80+ Thyroid Trial is to find out what effects (good and bad) thyroxine replacement has in older people with subclinical hypothyroidism. In total 150 Dutch participants will participate in the IEMO 80+ Thyroid Trial.

**2. What drug will be investigated?**

We investigate the effect of synthetic thyroid hormone (Levothyroxine) in older persons with subclinical hypothyroidism. This thyroid hormone is given by tablet orally (by mouth). We will compare the effects of the thyroid hormone tablets with effects of a placebo tablet. The placebo tablets contain no active drug, but are identical in look, taste and smell.

**3. How will the research study be conducted?**

You have been asked to take part in this study because your recent screening blood test has suggested that you may have subclinical hypothyroidism. In some persons subclinical hypothyroidism corrects over time, whilst in other persons a clear shortage of thyroid hormone is identified. For this reason the first phase (the selection phase) will determine whether you have a persistent subclinical hypothyroidism. Only when this is the case, are you eligible for the second phase of the study (the treatment phase).

If you agree to participate in the selection phase of the study we ask you to complete and sign the consent form and send this in the enclosed envelope to the study center in the Leiden University Medical Centre. The study center will return a laboratory form and will ask you to visit your general practice laboratory or hospital research facility for a screening visit within 2 weeks. In this screening visit a blood test will be performed to assess the levels of thyroid hormone. The results of this test will be send to the research center in the Leiden University Medical Centre. If you are unable to visit the laboratory, please contact the study center. They will arrange for a laboratory nurse to visit you at home.

If the results from the screening test indicate that you are not eligible for the treatment phase, the study center will inform you in writing with the screening results and an explanation. The screening result will be shared with your general practitioner or hospital specialist.

If the results from the screening tests are satisfactory you will be invited to take part in the treatment phase of the study. A research nurse will contact you with the screening results and to plan an appointment for a home visit. During this home visit the research nurse will explain the treatment phase of the research study and you may decide to participate in the treatment phase.

If you decide to take part, you will be asked to complete and sign a second consent form. After signing the form some medical questions will be asked (including general questions regarding health, medication use and quality of life) as well as some additional measurements taken (including blood pressure, heart rhythm and grip strength).

After the home visit a computer will randomly allocate you to either the levothyroxine or placebo treatment group. The chance of allocation to either group is equal (50%). The study drug will be taken daily, at least 30 min before breakfast, during a maximum of 2 years.

A research nurse will perform home visits at the start of the research study, after 6–8 weeks, after 12 months and after 24 months. We will ask you to visit the general practice laboratory before every home visit to assess thyroid hormone levels.

**4. What are the possible risks and benefits in participating?**

It is not certain whether you will gain personal benefit from participating in the research study. After all, the purpose of this research study is to assess whether treatment with levothyroxine provides important benefits. A potential benefit is that your thyroid function will be assesses regularly, both in the screening and treatment phase. For future older persons with subclinical hypothyroidism the research study may yield important information. The blood measurements taken will most likely not result in harmful effects.

**5. What happens if you decide not to participate in the research study?**

Your participation is entirely voluntary and you are not in any way obliged to take part. You decide whether you want to participate. If you decide not to participate, no further action is required, and you are not required to provide a reason for not participating. If you do decide to participate, you reserve the right to withdraw from the research study at any given time without providing a reason to do so. Whether you decide to participate or not will in no way affect the standard of care you receive or the relationship you have with your general practitioner or hospital specialist.

**6. Will the research study result in additional expenses/provide compensation?**

No. You will not be charged for expenses related to the study medication or blood tests. Participating in this research study will not affect your policy excess for medical insurance. No compensation is provided for participating in the research study.

**7. Further information?**

Should you have any additional questions regarding the research study you are welcome to contact the IEMO secretary, telephone 071–526 84 93, or the central study coordinator: The Institute for Evidence-Based Medicine in Old Age | IEMO.

Email: iemo_schildklierstudie@lumc.nl. More information can be found on the study website: www.iemoschildklierstudie.nl

For questions or problems you may also contact the independent general practitioner, Dr. Niels H. Chavannes, telephone 071–526 84 44, n.h.chavannes@lumc.nl. He is up-to-date with all proceedings of the trial, but is not involved with the conduct.

8. Appendices

1. Informed consent form.

2. envelope.

## Appendix 2: Patient Information leaflet for randomisation

**Thyroid hormone replacement for older persons with mild thyroid dysfunction**

*Several weeks ago your general practitioner or hospital specialist has invited you for a blood screening test. According to the test results you still have mild thyroid dysfunction (subclinical hypothyroidism). You are being invited to take part in a research study:*

***The IEMO 80+ Thyroid Trial***

*In this leaflet you’ll find detailed information regarding the research study. Please take your time to review the contents carefully and discuss these with your partner, friends or family. Should you have additional questions you are welcome to discuss these with your general practitioner, a researcher or the research nurse. Additionally you may contact the independent general practitioner, who knows a lot about the study, but is not involved with the conduct. Contact information can be found on the final page.*

**1. What is the purpose of the research study?**

Thyroid hormone has many important functions in the human body, for example supporting the correct function of the muscles, circulation, the brain and metabolism. When a shortage of thyroid hormone is present, bodily functions may not work optimally.

The results of one of your blood tests from the past suggest that you may have mild thyroid dysfunction (subclinical hypothyroidism). This is when thyroid hormone levels are within the normal laboratory limits, but signs are present that the body is urging the thyroid gland to work harder. This is usually a chance finding. This particular blood test result is common in older persons: of all persons aged 65 years and over 1 in 6 may have subclinical hypothyroidism. It is currently unknown whether it is beneficial to treat subclinical hypothyroidism with artificial thyroid hormone.

Having subclinical hypothyroidism is associated with slightly higher odds of developing cardiovascular diseases. Earlier small scale research demonstrated that treatment with synthetic thyroid hormone may provide beneficial effects on the circulation in older persons with subclinical hypothyroidism. Alternatively, synthetic thyroid hormone treatment may also result in unwanted side effects. Both the good and bad effects of thyroid hormone treatment have not been proven to this date.

The purpose of the IEMO 80+ Thyroid Trial is to find out what effects (good and bad) thyroxine replacement has in older people with subclinical hypothyroidism.

The research study has a specific aim to prevent cardiovascular diseases, and to improve the quality of life (for example by alleviating tiredness complaints), muscle strength and brain function. In total 150 persons in the Netherlands will take part in the IEMO 80+ Thyroid Trial. ‘IEMO 80+’ means that the research study is performed in persons aged 80 years and older. IEMO is an abbreviation for the Institute for Evidence-Based Medicine in Old Age, the institute responsible for coordinating the research study.

**2. What drug will be investigated?**

We investigate the effect of synthetic thyroid hormone (Levothyroxine) in older persons with subclinical hypothyroidism. This synthetic thyroid hormone is identical to the thyroid hormone produced by the human body and is the standard treatment when a definite shortage of thyroid hormone is identified in the blood. This thyroid hormone is given by tablet orally (by mouth). We will compare the effects of the thyroid hormone tablets with effects of a placebo tablet. The placebo tablets contain no active drug, but are identical in look, taste and smell. You will not be informed which of the two treatments you will receive. The study nurses are also unaware of your allocation.

You will start with 1 tablet, that will contain either 50 micrograms of levothyroxine (or 25 micrograms if your weight is below 50 kg or have a history of coronary strictures) or placebo. After 6–8 weeks all participants will have their blood hormone levels analysed. Based on these results a decision is made to change the treatment dose. If the laboratory results indicate a change in treatment dose is warranted a research nurse will explain this to you. From this point annual blood tests will monitor the response to the treatment (after 12 and 24 months).

**3. How will the research study be conducted?**

You have been asked to take part in this study because your recent screening blood test has suggested that you may have subclinical hypothyroidism.

If you agree to participate in the selection phase of the study we ask you to complete and sign the consent form and send this in the enclosed envelope to the study center in the Leiden University Medical Centre. We ask your permission to store additional blood samples for future research on blood and hereditary materials (DNA) that may influence the effects of thyroid hormone on bodily functions (such as the circulation, muscle strength, memory problems or frailty). The DNA will be isolated from the blood and stored for future research. This blood sample (equivalent to 8 teaspoons) will be taken during the first home visit. Other research not related to this research study will not have access to the stored blood and DNA samples and any information in the samples cannot be retraced to individual persons. If you object to the storage of blood and DNA you may choose not to participate in this particular portion of the trial.

After signing the consent form some medical questions will be asked (including general questions regarding health, medication use and quality of life) as well as some additional measurements taken (including blood pressure, heart rhythm and grip strength). This home visit will take approximately1 to 1.5 h.

After the home visit a computer will randomly allocate you to either the levothyroxine or placebo treatment group. The chance of allocation to either group is equal (50%). The study drug will be taken daily, at least 30 min before breakfast, during a maximum of 2 years.

A research nurse will perform home visits at the start of the research study, after 6–8 weeks, after 12 months and after 24 months. We will ask you to visit the general practice laboratory before every home visit to assess thyroid hormone levels.

**4. What is expected of you?**

If you decide to participate in the research study, you will be asked:

- To take between 1 and 3 tablets, once every day, in the morning
- To attend home visits with a research nurse for measurements
- To visit the general practice laboratory for thyroid hormone tests (after 6–8 weeks, after 12 months and after 24 months).

There are no lifestyle or dietary restrictions in this research study. Data from your medical records at the general practice office or hospital specialist will be collected. Your general practitioner or hospital specialist will be informed of your participation in the study.

**5. Are there other treatment options?**

There are no other treatment options for mild thyroid dysfunction (subclinical hypothyroidism).

**6. What are the possible side effects?**

Side effects from levothyroxine treatment are only rarely seen, particularly if blood tests are done regularly and adjustments to levothyroxine dosages are made to keep thyroid hormone levels in the normal range. If levothyroxine is prescribed in too high dosages there is a risk of side effects such as heart palpitations, tremors, sweating, feeling agitated, tiredness and shortness of breath and chest pains. Overmedication may cause leg swelling and there may be an increased risk of thinning of the bones (osteoporosis) and fractures.

If you suffer from epilepsy overmedication with thyroid hormone may result in an increased risk of seizures.

If you are on an anticoagulant it is possible that the dose may need to be adjusted to prevent your blood becoming too thin.

If you are allocated to the inactive tablets (placebo) there is a risk that the thyroid gland may slow down further, and they may develop symptoms of an underactive thyroid, including tiredness and lethargy. Should this develop during the research study the blood tests will identify an underactive thyroid and your general practitioner or hospital specialist will be consulted for starting treatment.

**7. What are the possible risks and benefits in participating?**

It is not certain whether you will gain personal benefit from participating in the research study. After all, the purpose of this research study is to assess whether treatment with levothyroxine provides important benefits. A potential benefit is that your thyroid function will be assesses regularly, both in the screening and treatment phase. For future older persons with subclinical hypothyroidism the research study may yield important information. The blood measurements taken will most likely not result in harmful effects.

**8. What happens if you decide not to participate in the research study?**

Your participation is entirely voluntary and you are not in any way obliged to take part. You decide whether you want to participate. If you decide not to participate, no further action is required, and you are not required to provide a reason for not participating. If you do decide to participate, you reserve the right to withdraw from the research study at any given time without providing a reason to do so. Whether you decide to participate or not will in no way affect the standard of care you receive or the relationship you have with your general practitioner or hospital specialist.

**9. What happens after the research study is finished?**

When the treatment phase of the study completes, the results will be analysed by the coordinating researchers. This process will last several months after the last participant has finished the duration of the study. You and your general practitioner will be informed in writing of your results during the study, and whether you were allocated to the levothyroxine or placebo group. Based on this information you and your general practitioner may discuss whether further treatment with levothyroxine is in your best interest.

**10. Are you insured during the research study?**

All participants of the research study are covered by insurance for potential damages resulting from the study, both during the study period and within 4 years of ending the study. At the end of this letter you will find the insured amounts, exceptions and contact information of the insurance agency.

**11. What if new information becomes available?**

The data from all study participants are reviewed at fixed time points by a specially installed independent commission. If the safety or quality of life of the participants is in jeopardy, this commission is entitled to stop the research study. The study team will contact you directly should this occur.

**12. What happens to the data collected?**

In this research study data from interviews, questionnaires and measurements will be collected. As well as blood sample analysis. All data and materials collected will be handled and stored confidentially. Only the lead investigator will have access to personal information. Unauthorized personnel will have no access to your data. The results from this research study will be published in scientific journals; the data will not be traceable to individual persons. Anonymised research data will be made available to the IEMO 80+ Thyroid Trial investigators.

**13. Will your general practitioner/hospital specialist be informed of study participation?**

We think it is important that your general practitioner or hospital specialist is informed when study medication is given. For this reason we will inform your general practitioner or hospital specialist in writing of your participation. A specific section of the consent form explains this in more detail. It is not possible to participate in the research study without this consent.

**14. Will the research study result in additional expenses/provide compensation?**

No. You will not be charged for expenses related to the study medication or blood tests. Participating in this research study will not affect your policy excess for medical insurance. No compensation is provided for participating in the research study.

**15. Who has reviewed the study?**

The Medical Ethics Committee from the Leiden University Medical Centre has reviewed and approved the research study.

**16. Further information?**

Should you have any additional questions regarding the research study you are welcome to contact the IEMO secretary, telephone 071–526 84 93, or the central study coordinator: The Institute for Evidence-Based Medicine in Old Age | IEMO.

Email: iemo_schildklierstudie@lumc.nl. More information can be found on the study website: www.iemoschildklierstudie.nl

For questions or problems you may also contact the independent general practitioner, Dr. Niels H. Chavannes, telephone 071–526 84 44, n.h.chavannes@lumc.nl. He is up-to-date with all proceedings of the trial, but is not involved with the conduct.

## Abbreviations

AE: Adverse event
eCRF: Electronic case report form
fT4: Free thyroxine
GP: General practitioner
IDMC: Independent Data Management Committee
IEMO: Institute for evidence-based medicine in old age
RCT: Randomised controlled trial
SAE: Serious adverse event
SCH: Subclinical hypothyroidism
ThyPRO: Thyroid-related quality of life patient-reported outcome
TRUST: Thyroid hormone replacement for untreated older adults with subclinical hypothyroidism - a randomised placebo controlled trial
TSH: Thyroid stimulating hormone

## References

[1] Jones DD, May KE, Geraci SA (2010). Subclinical thyroid disease. Am J Med.

[2] Garber JR, Cobin RH, Gharib H, Hennessey JV, Klein I, Mechanick JI, Pessah-Pollack R, Singer PA, Woeber KA, American Association of Clinical E (2012). Clinical practice guidelines for hypothyroidism in adults: cosponsored by the American Association of Clinical Endocrinologists and the American Thyroid Association. Endocr Pract.

[3] Biondi B, Cooper DS (2008). The clinical significance of subclinical thyroid dysfunction. Endocr Rev.

[4] Stott DJ, Rodondi N, Kearney PM, Ford I, Westendorp RGJ, Mooijaart SP, Sattar N, Aubert CE, Aujesky D, Bauer DC (2017). Thyroid hormone therapy for older adults with subclinical hypothyroidism. N Engl J Med.

[5] Devdhar M, Drooger R, Pehlivanova M, Singh G, Jonklaas J (2011). Levothyroxine replacement doses are affected by gender and weight, but not age. Thyroid.

[6] Flynn RW, Bonellie SR, Jung RT, MacDonald TM, Morris AD, Leese GP (2010). Serum thyroid-stimulating hormone concentration and morbidity from cardiovascular disease and fractures in patients on long-term thyroxine therapy. J Clin Endocrinol Metab.

[7] Gussekloo J, van Exel E, de Craen AJ, Meinders AE, Frolich M, Westendorp RG (2004). Thyroid status, disability and cognitive function, and survival in old age. JAMA.

[8] Rodondi N, den Elzen WP, Bauer DC, Cappola AR, Razvi S, Walsh JP, Asvold BO, Iervasi G, Imaizumi M, Collet TH (2010). Subclinical hypothyroidism and the risk of coronary heart disease and mortality. JAMA.

[9] Jansen SW, Akintola AA, Roelfsema F, van der Spoel E, Cobbaert CM, Ballieux BE, Egri P, Kvarta-Papp Z, Gereben B, Fekete C, et al. Human longevity is characterised by high thyroid stimulating hormone secretion without altered energy metabolism. Sci Rep. 2015;5 10.1038/srep11525 PMC4473605 26089239

[10] Stott DJ, Gussekloo J, Kearney PM, Rodondi N, Westendorp RG, Mooijaart S, Kean S, Quinn TJ, Sattar N, Hendry K (2017). Study protocol; thyroid hormone replacement for untreated older adults with subclinical hypothyroidism - a randomised placebo controlled trial (TRUST). BMC Endocr Disord.

[11] Folstein MF, Folstein SE, McHugh PR (1975). “Mini-mental state”. A practical method for grading the cognitive state of patients for the clinician. J Psychiatr Res.

[12] Watt T, Hegedus L, Groenvold M, Bjorner JB, Rasmussen AK, Bonnema SJ, Feldt-Rasmussen U (2010). Validity and reliability of the novel thyroid-specific quality of life questionnaire, ThyPRO. Eur J Endocrinol.

[13] Rabin R, Gudex C, Selai C, Herdman M (2014). From translation to version management: a history and review of methods for the cultural adaptation of the EuroQol five-dimensional questionnaire. Value Health.

[14] Watt T, Bjorner JB, Groenvold M, Cramon P, Winther KH, Hegedus L, Bonnema SJ, Rasmussen AK, Ware JE, Feldt-Rasmussen U (2015). Development of a short version of the thyroid-related patient-reported outcome ThyPRO. Thyroid.

[15] Smith A (1968). The symbol digit modalities test. A neuropsychological test for economic screening of learning and other cerebral disorders. Learn Disord.

[16] Mahoney FI, Barthel DW (1965). Functional Evaluation: The Barthel index. Md State Med J.

[17] Quinn TJ, Langhorne P, Stott DJ (2011). Barthel index for stroke trials: development, properties, and application. Stroke.

[18] George LK, Fillenbaum GG (1985). OARS methodology. A decade of experience in geriatric assessment. J Am Geriatr Soc.

[19] Bohannon RW (2009). Measurement of gait speed of older adults is feasible and informative in a home-care setting. J Geriatr Phys Ther (2001).

[20] Atkinson MJ, Kumar R, Cappelleri JC, Hass SL (2005). Hierarchical construct validity of the treatment satisfaction questionnaire for medication (TSQM version II) among outpatient pharmacy consumers. Value Health.

[21] Winther KH, Cramon P, Watt T, Bjorner JB, Ekholm O, Feldt-Rasmussen U, Groenvold M, Rasmussen AK, Hegedus L, Bonnema SJ (2016). Disease-specific as well as generic quality of life is widely impacted in autoimmune hypothyroidism and improves during the first six months of levothyroxine therapy. PLoS One.

[22] Villar HC, Saconato H, Valente O, Atallah AN (2007). Thyroid hormone replacement for subclinical hypothyroidism. Cochrane Database Syst Rev.

