# Supplementary material for: Study protocol: a randomised controlled trial on the clinical effects of levothyroxine treatment for subclinical hypothyroidism in people aged 80 years and over
Source: BMC Endocr Disord. 2018 Sep 19;18:67. doi: 10.1186/s12902-018-0285-8 (PMC6146605; doi:10.1186/s12902-018-0285-8)
Supplement: Supplementary file 1 — Statistical analysis plan. (PDF 114 kb) [file 12902_2018_285_MOESM1_ESM.pdf]

IEMO80+

Statistical Analysis Plan

Final Analysis

Study Title:	IEMO 80-plus thyroid trial		
Short Title:	IEMO80+		
EudraCT Number:	2012-004160-22		
Funded by:	ZonMW programme Evidence-Based Medicine in Old Age (EMO).<br>ZonMW project number: 627001001		
Protocol Version:	9	Date:	14/06/2016
SAP Version:	v1.0	Date:	02/05/2018

		Signature	Date
Prepared by:	Dr Martina Messow<br>Consultant Statistician<br>Robertson Centre for Biostatistics<br>University of Glasgow	[illegible]	08/05/2018
Approved by:	Prof Ian Ford<br>Director<br>Robertson Centre for Biostatistics<br>University of Glasgow	[illegible]	08/05/2018
	Dr Simon Mooijaart<br>Director of IEMO<br>Department of Gerontology and Geriatrics<br>Leiden University Medical Center	[illegible]	10/05/2018

CONTENTS

1. Introduction 3
   1.1. Study Background 3
   1.2. Study Objectives 3
   1.3. Study Design 3
   1.4. Sample Size and Power 3
   1.5. Study Population 3
      1.5.1. Inclusion Criteria 3
      1.5.2. Exclusion Criteria 3
   1.6. Statistical Analysis Plan (SAP) 4
      1.6.1. SAP Objectives 4
      1.6.2. General Principles 4
      1.6.3. Current Protocol 5
      1.6.4. Deviations To Those Specified In Study Protocol 6
      1.6.5. Additional Analyses To Those Specified In Study Protocol 6
      1.6.6. Software 6
2. Analysis 6
   2.1. Study Populations 6
   2.2. Data pooling 7
   2.3. Study Status and Consort diagram 7
   2.4. Visit Attendance 8
   2.5. Baseline Characteristics 8
   2.6. Efficacy Outcomes at 12 months 9
      2.6.1. Primary Outcome 9
      2.6.2. Secondary Outcomes 9
      2.6.3. Sensitivity analyses 9
      2.6.4. Subgroup analyses 10
   2.7. Efficacy outcomes at end of study 10
   2.8. Efficacy Outcomes at Other Timepoints 11
   2.9. Safety Outcomes 11
      2.9.1. Study Treatment 12
      2.9.2. Withdrawals from the study 12
      2.9.3. Serious adverse events 12
      2.9.4. Concomitant medications 12
      2.9.5. Other safety data 12
3. Document History 13
4. Tables and Figures 13
5. Listings 13

1. Introduction

1.1. Study Background

Subclinical hypothyroidism (SCH) is a common finding in older people across Europe, and is a likely contributor to multiple problems in older age. There is the potential for multisystem benefits from treatment of SCH with Levothyroxine.

1.2. Study Objectives

To test the efficacy of thyroxine replacement for subclinical hypothyroidism (SCH) in adults aged 80 years and older.

1.3. Study Design

Randomised double-blind placebo-controlled parallel group trial.

1.4. Sample Size and Power

The study aims to have 291 patients available for the final analysis. As the trial aims to combine analyses with the TRUST study, which was expected to contribute 146 patients aged 80 years or older, the aim is to include 145 patients in the IEMO80+ study. For more detail about the sample size calculation see protocol section 4.4.

1.5. Study Population

Potential subjects will be identified from clinical laboratory databases as having biochemical features consistent with SCH, (thyroid stimulating hormone [TSH] of ≥4.6 and ≤19.9 mU/L plus free thyroxine levels within the laboratory reference range, measured on at least two occasions at least three months apart).

1.5.1. Inclusion Criteria

Community-dwelling subjects aged ≥80 years with SCH, diagnosed on the basis of elevated TSH levels (≥4.6 and ≤19.9 mU/L) and free thyroxine levels (fT4) within the laboratory reference range, measured on a minimum of two occasions at least 3 months apart (in Switzerland there was a minor variation the main study protocol - patients required only one measure of fT4 before recruitment).

1.5.2. Exclusion Criteria

- Subjects currently on (anti)thyroid drugs, amiodarone or lithium;

- Recent thyroid surgery or radio-iodine therapy;
- Grade IV NYHA heart failure;
- Prior clinical diagnosis of dementia;
- Recent hospitalisation for major illness;
- Recent acute coronary syndrome;
- Acute myocarditis or acute pancarditis;
- Untreated adrenal insufficiency or adrenal disorder;
- Terminal illness;
- Participants with rare hereditary problems of galactose intolerance, the Lapp lactase deficiency or glucose-galactose malabsorption;
- Subjects who are participating in ongoing RCTs of therapeutic interventions (including CTIMPs);
- Plan to move out of the region in which the trial is being conducted within the next 2 years.

1.6. Statistical Analysis Plan (SAP)

1.6.1. SAP Objectives

The objective of this SAP is to describe the statistical analyses to be carried out for the Final Analysis of the IEMO80+ study.

1.6.2. General Principles

Where applicable, all analyses will be carried out separately for

- All patients in IEMO80+
- All patients in TRUST aged 80 or over
- All patients in IEMO80+ and all patients in TRUST aged 80 or over combined
- All patients in TRUST aged under 80
- All patients combined

Data will be summarised overall and by treatment group. Continuous variables will be summarised as number of observed values, number of missing values, mean and standard deviation, median and interquartile range and minimum and maximum. Categorical data will be summarised as number of observed values, number of missing values, number and percentage in each category.

Continuous efficacy outcomes involving measurement at follow-up and baseline will be analysed as change from baseline at each time point comparing treatment groups and adjusting for stratification variables and baseline levels of the same variable using linear regression. In addition, data

items measured at more than one follow-up time will be analysed using repeated measures regression analyses and in terms of the final assessment for each participant.

Continuous efficacy outcomes measured at final follow-up only will be compared between treatment groups using linear regression adjusting for stratification variables.

For all efficacy outcomes measured at baseline and final visit, additional exploratory analyses that adjust for time from baseline to final visit will be carried out.

Distributions of the residuals will be reviewed and will be taken into consideration in assessing whether or not additional analyses based on transformations should be carried out.

When calculating ThyPRO scores, valid raw total scores containing missing items will be scaled so that the maximum possible score is maintained.

Time-to-event outcomes will be compared between groups using Cox proportional hazards regression models adjusting for stratification variables. Time to event curves will be based on the Kaplan-Meier method.

Stratification variables are

- Country
- Gender
- Starting dose of levothyroxine

All efficacy analyses will be carried out on the intention to treat (ITT) population. Safety analyses will be carried out on the ITT population. The main analyses will be modified ITT based on participants with data on the outcome of interest. These analyses will be supported with sensitivity analyses using mixed effects models and multiple imputation. Primary and secondary analyses will be repeated on the per protocol population as exploratory analyses.

The two-sided significance level for each primary outcome is 0.025.

1.6.3. CURRENT PROTOCOL

At the time of writing, the current protocol for the IEMO80+ study is version 9, dated 14/06/2016.

Future amendments to this protocol will be reviewed for their impact on this SAP, which will be updated only if necessary. If no changes are required to this SAP following future amendments to the study protocol, this will be

documented as part of the Robertson Centre Change Impact Assessment processes.

1.6.4. Deviations To Those Specified In Study Protocol

Analyses of haemoglobin data, Treatment Satisfaction Questionnaire for Medications (TSQM) and Questionnaire about arthrosis will be described separately.

1.6.5. Additional Analyses To Those Specified In Study Protocol

No additional analyses currently planned. Any additional analyses will be documented appropriately.

1.6.6. Software

Analyses will be carried out using R for Windows v3.0.1, SAS for Windows v9.3, or higher versions of the programs.

2. Analysis

2.1. Study Populations

The intention-to-treat (ITT) population consists of all participants randomised excluding those randomised in error.

Modified ITT (mITT) populations will include ITT participants who have data for the outcome of interest. For analyses at the 12 month visit to be valid, they must have been conducted at 12 months ± 31 days after randomisation.

Per-protocol (PP) populations consists of all participants in the ITT population who

- have data for the outcome variable of interest;
- are on treatment at the time of the data points for the analysis being conducted, i.e. they have not withdrawn from treatment and, if they are in the active treatment group, they have not been down-titrated to 0;
- have had their 12 month visit at 12 months ± 31 days after randomisation;
- do not have any other major protocol violation identified prior to database lock.

Reasons for exclusion from PP populations will be summarised.

2.2. DATA POOLING

Data from the IEMO80+ study will be pooled with data from the TRUST study in different ways, which will result in the following populations:

- IEMO80+ patients only (IEMO)
- TRUST patients aged 80 or older (TRUST80+)
- IEMO80+ patients and TRUST patients aged 80 years or older (80+)
- TRUST patients aged less than 80 (TRUST80-)
- All IEMO80+ and TRUST patients (ALL)

mITT and PP populations will be defined within each of these populations.

2.3. STUDY STATUS AND CONSORT DIAGRAM

A study status table will be created describing the following numbers:

- Number screened
- Number excluded, among those:
    - Number who did not meet inclusion / exclusion criteria and among these the number who had reversion of TSH level to <4.6 mIUI/l
    - Number who did not proceed to randomisation
- Number randomised
- Number included in 12-month follow-up, by randomised treatment group
- Number with primary outcome data, by randomised treatment group
- Number included in follow-up past 12 months, by randomised treatment group
- Numbers who are dead or have withdrawn consent at 12-month follow-up, by randomised treatment group
- Numbers who are dead or have withdrawn consent at final follow-up, by randomised treatment group

The table will contain the numbers included in the main analysis of the primary outcome for both the ITT and PP populations. It will also contain the

respective numbers for all patients in TRUST aged 80 or older and IEMO80+ separately.

This table will be used to create a CONSORT diagram for the trial.

2.4. VISIT ATTENDANCE

For each visit, the number and percentage who attended the visit, missed the visit, had completed the study before the visit, had withdrawn from follow-up or had died before the visit will be reported. A visit will be considered as attended if any measurement or participant reported data for this visit are available. A visit will be considered as missed if there are no data for this visit and the participant has not died, not completed the study and not withdrawn from follow-up. Similar tables will be created for TRUST patients aged 80 and older.

2.5. BASELINE CHARACTERISTICS

The following baseline characteristics will be summarised overall and by treatment group (an additional tabulation will be created for ITT participants by inclusion/exclusion from the modified ITT analysis for the primary outcome):

- Age (continuous), gender, ethnicity, smoking history, alcohol consumption, living arrangements, height;
- cardiovascular risk factors (MI, angina, stroke, TIA, heart failure, PVD, revascularisation, AF, hypertension, diabetes);
- medical history (epilepsy, dementia, osteoporosis, hereditary galactose intolerance, other major illness);
- Mini Mental State Exam (MMSE) score (IEMO80+ only);
- laboratory measures; TSH and fT4 at baseline;
- single lead ECG results (heart rate, cardiac rhythm);
- falls (all questions) (IEMO80+ only);
- baseline values of outcome measures (see Section 2.5.2.);
- use of concomitant medications, using the WHO ATC classification system level 1 and 2, ordered by ATC code.

These summaries will be produced for the mITT populations.

2.6. EFFICACY OUTCOMES AT 12 MONTHS

2.6.1. PRIMARY OUTCOME

Disease-specific quality of life and symptom burden will be analysed as follows in the mITT and PP populations:

- Change from baseline in ThyPRO hypothyroid symptoms score. This outcome will be analysed in the modified ITT population as

  The estimated treatment effect (p-value and 95%CI) in a linear regression model predicting change from baseline to 12 month visit in ThyPRO hypothyroid symptoms score with the following covariates: randomised treatment, ThyPRO hypothyroid symptoms score at baseline and stratification variables.

- Change from baseline in ThyPRO fatigue score. This outcome will be analysed analogously.

- Statistical significance for each primary outcome requires $P \leq 0.025$.

The primary analysis will be on the mITT 80+ population, the analyses in all other populations are secondary analyses.

2.6.2. SECONDARY OUTCOMES

The following secondary outcomes will be analysed at 12 months in the mITT and PP populations

- General QoL (EQ-5D)
- Handgrip strength
- TSH
- fT4
- Blood pressure
- Weight, waist circumference and BMI
- Falls (any falls) (IEMO80+ only).

These variables will be analysed as for the primary outcomes. For falls outcome, logistic regression will be used.

2.6.3. SENSITIVITY ANALYSES

Sensitivity analysis for the mITT analyses for the primary and secondary outcomes at 12 months will be carried out using a) multiple imputation of missing values using a model containing age, sex, baseline TSH and any

available outcome measurements recorded prior to 12 months and b) using mixed model repeated measures analysis.

2.6.4. Subgroup analyses

The primary and secondary outcomes at 12 months will also be analysed in the mITT populations in the following subgroups by adding the subgroup variable and its interaction with treatment group:

- Study (IEMO80+ / TRUST) (80+ and ALL population only)
- Age (<80 / ≥80) (ALL population only)
- Gender (Male/Female)
- Baseline TSH
    - <10 / ≥10
    - <7 / 7-9.99 / ≥10

In addition, continuous interactions with age at randomisation and baseline TSH will be analysed analogously.

2.7. Efficacy outcomes at end of study

The following measurements will be assessed at baseline and the end of study visit:

- ThyPRO hypothyroid symptoms and fatigue scores
- General QoL (EQ-5D)
- Handgrip strength
- Cognitive function (letter digit coding test (LDCT),
- Functional ability (Barthel index, IADL)
- Gait speed over 12 m (IEMO80+ only)
- TSH
- fT4
- Blood pressure
- Weight, waist circumference and BMI
- Living independently
- Falls (any falls) (IEMO80+ only)

These outcomes will be analysed as for the primary outcome, apart from falls which will be analysed as specified for the 12 month analysis. An additional

sensitivity analysis will be carried out adjusting for the time from baseline to the measurement assessment.

The following outcomes will be assessed at the end of study as described below:

- Comprehensive thyroid quality of life assessment ThyPRO39 at the end of study visit

  The estimated treatment effect (p-value and 95%CI) in a linear regression model predicting end of study ThyPRO39 score with the following covariates: randomised treatment and stratification variables.

- Fatal and non-fatal cardiovascular events (confirmed by Endpoint Adjudication Committee)

  The estimated treatment effect (p-value and 95%CI) in a Cox proportional hazards models containing randomised treatment group and stratification variables.

- Fatal cardiovascular events (confirmed by Endpoint Adjudication Committee). This outcome will be analysed analogously.
- All-cause mortality. This outcome will be analysed analogously.

2.8. Efficacy Outcomes at Other Timepoints

All outcomes assessed post randomisation at timepoints other than baseline, 12 months and end of study will be investigated as change from baseline at these timepoints, analysed as for the primary outcome.

In addition, change from baseline for outcomes analysed at two or more post randomisation visits will be analysed using repeated measures ANOVA models with general covariance structures, first containing only treatment group, study visit, the baseline value of the outcome being assessed and stratification variables and then including a treatment by study visit interaction.

Results at each time point and changes from baseline will also be presented graphically as means and 95% CIs at each time point.

2.9. Safety Outcomes

All safety analyses will be done in all ITT populations.

2.9.1. STUDY TREATMENT

Starting dose and dose just before 2, 12, 24, 36 month visits, current dose at final visit and dose at the end of the titration phase in year 1 will be summarised overall and by treatment group.

Time to permanent withdrawal from study treatment (excluding deaths) will presented using Kaplan-Meier time-to-event curves and compared using log-rank statistics. Reasons for permanent withdrawal from study treatment will be tabulated overall and split by treatment group.

2.9.2. WITHDRAWALS FROM THE STUDY

Numbers withdrawn from the study and the main reasons for withdrawal will be summarised overall and by treatment group. Kaplan-Meier plots for time to withdrawal will be produced.

2.9.3. SERIOUS ADVERSE EVENTS

The number and characteristics of SAEs will be summarised. The number and percentage of participants experiencing at least one SAE will be summarised overall and by MedDRA System Organ Class and preferred term. These summaries will be repeated for SAEs at least possibly related to study medication, and for fatal events. Tabulations will be sorted by the MedDRA SOC term order and by preferred term order with in SOCs.

The incidence of other safety outcomes of particular interest (hypothyroidism, atrial fibrillation, heart failure, fractures), will be summarised separately.

Kaplan-Meier plots by treatment for the time to the first occurrence of each safety outcome of particular interest will be produced. Time to event will be compared between groups using log-rank tests. If there are sufficient events, Cox proportional hazards regression adjusting for stratification variables will be used.

2.9.4. CONCOMITANT MEDICATIONS

The number and percentage of participants receiving at least one concomitant medication will be summarised by ATC codes level 1 and 2.

2.9.5. OTHER SAFETY DATA

- ThyPRO hyperthyroid symptoms will be summarised at each time point, overall and by treatment group. Change in ThyPRO hyperthyroid symptoms will be compared between treatment groups in the same way as the primary analysis.
- A listing of deaths will be provided.

3. DOCUMENT HISTORY

This is version 1.0 (initial creation) of the SAP for the final analysis for IEMO80+, dated 02/05/2018.

4. TABLES AND FIGURES

The layout of the tables and figures will be finalised based on tables created using dummy treatment codes prior to database lock.

5. LISTINGS

All SAEs will be listed. This listing will be generated for all IEMO80+ and TRUST patients, displaying study and age group for each one, ordered by study, age group and subject ID.
