# Supplementary material for: Study protocol: a randomised controlled trial on the clinical effects of levothyroxine treatment for subclinical hypothyroidism in people aged 80 years and over
Source: BMC Endocr Disord. 2018 Sep 19;18:67. doi: 10.1186/s12902-018-0285-8 (PMC6146605; doi:10.1186/s12902-018-0285-8)
Supplement: Supplementary file 2 — Participant consent form for screening. (PDF 68 kb) [file 12902_2018_285_MOESM2_ESM.pdf]

IEMO Schildklier Studie

Informed Consent Form 1

Screening phase

IEMO eCRF no:

The IEMO 80+ Thyroid Trial

- I've read the information letter. I was able to ask additional questions. My questions have been answered satisfactorily. I have had enough time to consider my participation.
- I understand that my participation is completely voluntary and that I am free to withdraw at any time, without giving any reason.
- I agree that my GP and/or treating specialist are informed about my study
- I agree that my GP and/or treating specialist are informed about the results of the blood tests
- I agree to using my data for the aims described in the information letter
- I agree with participating in the selection phase of this study.

Last name and initials: ………………………………………………………...

Address: ………………………………………………………….

Postal code: ………………………………………………………….

City: ………………………………………………………….

Sex : **… male … female**

Date of birth: ………………………………………………………...

Date: …. …. …. **Phone:** ………………

Signature: ………………………………………………………..
