# Supplementary material for: Study protocol: a randomised controlled trial on the clinical effects of levothyroxine treatment for subclinical hypothyroidism in people aged 80 years and over
Source: BMC Endocr Disord. 2018 Sep 19;18:67. doi: 10.1186/s12902-018-0285-8 (PMC6146605; doi:10.1186/s12902-018-0285-8)
Supplement: Supplementary file 3 — Participant consent form randomisation. (PDF 83 kb) [file 12902_2018_285_MOESM3_ESM.pdf]

IEMO Schildklier Studie

Informed Consent form 2
Treatment phase

IEMO eCRF no:

The IEMO 80+ Thyroid Trial

- I have read the information letter
- I was able to ask additional questions
- My questions have been answered satisfactorily
- I have had enough time to consider my participation
- I understand that my participation is completely voluntary
- I understand that I am free to withdraw at any time, without giving any reason.
- I agree that my GP and/or treating specialist are informed about my study participation
- I agree that my GP and/or treating specialist are informed about the results of the blood tests
- I agree that, if necessary, the thrombosis service is informed about my study participation
- I agree to using my data for the aims described in the information letter
- I agree to share my anonymized data with the IEMO 80+ Thyroid Trial researchers
- I agree that medical information from my GP or treating specialist relevant to the study may be requested
- I agree that my study data are stored for a maximum of 15 years after the end of the study
- I agree that the IEMO 80+ thyroid trial researchers can request my cause of death information from the central bureau of statistics to be used for this study
- I do/don't* agree have additional blood taken at start of study and after one year and store this for maximal 15 year in order to use it for new studies
- I do/don't* agree to store my DNA in order to do future research
- I agree with participating in this study

* Strike out what does not apply.

Name participant: ………………………………………………………………..

Date of birth participant: ………………………………………………………………..

Signature: ………………………………………………………………..

Date: …. …. …. **Phone:** …………………….…..

This part is for the researcher only

I declare that I have informed the participant completely about the study

If information becomes available during the study that could influence the consent of the participant, I will inform the participant timely.

Name researcher (or representative):

Signature:

Date: __ / __ / __

Additional information has been provided by (if applicable):

Name:

Function:

Signature:

Date: __ / __ / __
